# Gender-Specific Differences in Self-Care, Treatment-Related Symptoms, and Quality of Life in Hemodialysis Patients

**DOI:** 10.3390/ijerph182413022

**Published:** 2021-12-10

**Authors:** Claudia Lerma, Larissa I. Lima-Zapata, Jorge A. Amaya-Aguilar, Itzel Leonardo-Cruz, Monica Lazo-Sánchez, Luis A. Bermúdez, Héctor Pérez-Grovas, Abel Lerma, Julio César Cadena-Estrada

**Affiliations:** 1 Department of Electromechanical Instrumentation, National Institute of Cardiology Ignacio Chávez, Mexico City 14080, Mexico; lermag@unam.mx (C.L.); larissalimazap94@gmail.com (L.I.L.-Z.); 2 Department of Nursing Research, National Institute of Cardiology Ignacio Chávez, Mexico City 14080, Mexico; skymanzeus@gmail.com (J.A.A.-A.); jitziunam@gmail.com (I.L.-C.); lzmonimar@gmail.com (M.L.-S.); 3 Centro Estatal de Hemodiálisis, Instituto Estatal de Cancerología, Colima 28000, Mexico; lbermudeza@hotmail.com; 4 Department of Nephrology, National Institute of Cardiology Ignacio Chávez, Mexico City 14080, Mexico; hpgrovas@gmail.com; 5 Institute of Health Sciences, Universidad Autónoma del Estado de Hidalgo, San Juan Tilcuautla, Pachuca 42160, Mexico; abel_lerma@uaeh.edu.mx

**Keywords:** end-stage renal disease, gender role, hemodialysis symptoms, quality of life, self-care

## Abstract

Gender and sex differences affect women with kidney failure (KF) negatively at all stages of the disease. This study assessed gender differences in self-care, hemodialysis symptoms, and quality of life in a sample of 102 adult KF patients treated with hemodialysis, from two clinical centers in Mexico. Self-care agency, quality of life, and the symptoms related to hemodialysis were evaluated through questionnaires, and sociodemographic and laboratory variables were obtained from the clinical records. Compared to male patients, female patients reported similar self-care, lower quality of life subscales (symptoms, physical functioning, pain, and overall health), and higher prevalence and intensity of hemodialysis symptoms. There were gender differences regarding the correlation between self-care and quality of life, symptoms intensity, and symptoms prevalence. In conclusion, women with KF treated with hemodialysis perceived a higher impact of hemodialysis and reported a lower quality of life than men. Despite having a similar self-care agency, the self-care correlations with quality of life and hemodialysis symptoms appeared different between men and women treated with chronic hemodialysis. Such differences may be important in future nursing interventions to improve self-care and quality of life among KF patients.

## 1. Introduction

Chronic renal disease affects 10% of the global population. In Latin America, there is an increase in the prevalence of kidney failure (KF), which is associated with an accelerated incidence of patients with substitute renal treatment, including hemodialysis (HD) [1]. The quality of life (QoL) can decrease considerably in KF patients, which increases their mortality risk [2]. The aspects that are more affected in the QoL of KF patients are renal disease overload, physical function, social support, and overall health [3]. This points out the need for more planned and integral care from the team of health professionals for these patients.

The assessment of QoL includes several symptoms [4], but there is a questionnaire that achieves an integral evaluation of symptoms associated with HD [5]. Patients undergoing maintenance HD use to experience diverse physical symptoms (e.g., fatigue and lack of appetite) and emotional symptoms related to HD (e.g., depression and anxiety) [6]. Even though some symptoms can have an important severity [5], HD symptoms are usually underestimated by the health staff [7], and patients do not receive treatment for those symptoms [8]. The prevalence and severity of symptoms are associated with lower QoL [5]. Non-pharmacological strategies to improve QoL that consider patients’ symptoms are focused on some defined type of symptoms, either related to physical functioning (e.g., improving muscle strength through exercise) [9] or psycho-emotional (e.g., decreasing depressive and anxious symptoms through psychological interventions) [10]. Those interventions are effective for their therapeutic target [11] and involve some degree of active patient’s participation, but they often do not consider the patient’s perspective about their own ability to cope with multiple stressors inherent to their disease [12]. This could be crucial for their capability to successfully respond to the complex challenges of KF [12].

There are gender and sex differences that negatively affect women with KF from the initial diagnosis through access to a substitute renal treatment [13,14], including renal transplant [15]. Some studies have shown lower QoL among women in HD [16]. Gender differences in self-care of renal patients have been studied scarcely. A study observed that women had similar self-care capacity than men, but they showed higher scores in self-efficacy [17]. It has also been reported that women in HD have better self-care efficacy of their arterio–venous fistula than men [18]. However, not all studies link the gender of HD patients with diverse self-efficacy aspects such as compliance to treatment [19].

Considering that the association between HD symptoms, self-care, and QoL has been studied scarcely, particularly from the perspective of gender, the aims of this exploratory work were: (i) to assess gender differences of self-care, HD symptoms, and QoL in KF patients treated with HD and (ii) to identify the associations between self-care, quality of life, and HD symptoms in these patients.

## 2. Materials and Methods

### 2.1. Study Participants

This exploratory cross-sectional study included 102 adult KF patients (55 men and 47 women) who were enrolled from two HD clinics located at Mexico City, during the first semester of 2017. All patients were adults, treated for at least a year with HD and had preserved cognitive function. This research complies with the principles described in the Declaration of Helsinki, and the study protocol was approved by the Research and Ethics Committees of our institution (protocol number 17-1015). The completed STROBE checklist is in the Supplementary Materials (File S1) [20]. The sampling method was non-probabilistic. Since there are no previous studies about the correlation of the three main studied variables, we considered a sample size of 100 patients for the study, considering that a Pearson’s correlation ≥0.35 between any pair of the studied variables would be significant with an alpha error of 0.05 and a statistical power of 95%.

### 2.2. Study Protocol

All eligible patients were invited during a personal interview; those interested in participating read the informed consent and were able to express their questions regarding the study. After written consent was obtained, each patient received a copy of the questionnaires and a detailed explanation on how to fill them in. Patients were able to fill the questionnaires in a well illuminated and ventilated room, in the quiet presence of a study researcher ready to answer any question. Clinical and laboratory results were obtained from the clinical records. Patients with incomplete data were eliminated from the study.

### 2.3. Questionnaires

Self-care was assessed with a Spanish language questionnaire of 43 items comprising three self-care agency dimensions development, operability, and adequacy [4]. The total self-care score was calculated in percentual units with the following equation: total self-care = (self-care score − 24) × 100)/96. The QoL was evaluated with the Kidney Disease Quality of Life Instrument (KdQoL) Version 1.3, which assesses QoL in KF patients with a renal replacement treatment [21]. The perceived HD symptoms were evaluated with the dialysis symptom index (DSI), which contains 30 items that assess the prevalence and intensity of physical and emotional symptoms associated with HD [5]. The Spanish language translation has reliability (Cronbach’s alpha) of 0.889. A detailed description of the reliability analysis is in the Supplementary Materials (File S2). For this questionnaire, a correlation between HD symptoms and QoL in HD patients has been documented [6].

### 2.4. Statistical Analysis

Nominal variables are shown as absolute value and percentage and were compared regarding the CAC level by a chi-squared test. For ordinal variables, a Kolmogorov–Smirnov test was applied to verify normal distribution. Variables with normal distribution are shown as a mean ± standard deviation and were compared between groups with a Student’s *t*-test. Other variables are shown as median (percentile 25–percentile 75) and were compared between groups with a Mann–Whitney U test. The correlations between the self-care and QoL questionnaires were calculated with the Spearman method. An analysis of variance was used to identify differences in the self-care score and the categorical variables compared by gender. The analysis was performed with the Statistical Package for Social Sciences (SPSS) version 21.0. A *p*-value < 0.05 was considered statistically significant.

## 3. Results

Table 1 shows anthropomorphic characteristics, comorbidities, and laboratory results from the 102 study participants. Compared to men, women had lower serum hemoglobin, creatinine, blood urea nitrogen, uric acid, and sodium, as well as presented more cases with autoimmune diseases and allergies. Women also included more patients with immune disease as KF etiology, while in men, essential hypertension was a more frequent KF etiology. There were no significant differences among the other characteristics.

Table 2 shows that there were no significant differences between gender on demographic characteristics.

The results of self-care, HD symptoms, and QoL are shown in Table 3. There were no differences by gender on the total self-care score. Compared to men, women had higher scores for prevalence and intensity of HD symptoms and lower QoL scores for the sub-scales of symptoms, physical functioning, pain, and overall health. There were no significant differences for the other QoL subscales or the total QoL scale.

Table 4 shows that self-care had a positive correlation in both genders with QoL (in the total scale and the subscales of physical functioning and fatigue/energy). Only in women, self-care had a positive correlation with QoL in the sub-scales of disease effect, disease load, cognitive functioning, social support, patient satisfaction, and emotional state. On the other side, only men showed a positive correlation between self-care and QoL in the sub-scale of sleep. There was no correlation between self-care and the other QoL subscales. The correlation analysis between HD symptoms and QoL showed that both genders had negative correlations with HD symptoms (prevalence and intensity) and QoL (total score and subscales of symptoms, effect, load, cognitive function, social interaction quality, pain, emotional state, and emotional role, social function, and fatigue/energy). Only in women, the prevalence and intensity of symptoms were negatively correlated with QoL in the physical role and overall health sub-scales. There was no correlation between HD symptoms and the other QoL sub-scales (work status, sexual functioning, positive interaction with the health staff, and patient satisfaction).

The associations between self-care, sociodemographic variables, and laboratory results were assessed with a Spearman correlation analysis (Table 5). There was no correlation between self-care and the evaluated variables, except for the transportation time from home to the HD clinic, which had a positive correlation with self-care in all patients but was not significant in the groups separated by gender.

Table 6 shows that for nominal variables, only in the group of women, diabetic patients had a lower self-care score than non-diabetic women, and patients treated with hemodiafiltration had a higher self-care score than those treated with HD. There were no differences by gender for the other nominal variables.

## 4. Discussion

Our results show that in most patients (both men and women) a higher self-care was associated with a higher QoL on physical functioning and fatigue/energy. Similar results from Trask et al. [22] showed that with higher self-care, the QoL was better and that an increase in HD knowledge by the patients and self-care promotion in their treatment decreased their one-year mortality. A previous report showed that patients in chronic HD consider physical activity as an important self-care factor to maintain their health, but at the same time, they consider that an important barrier to practicing physical activity is the fatigue attributed both to the disease and to the HD treatment [23]. The present study shows that, although there was a significant association between physician functioning and self-care for both genders (Table 4), female patients reported a greater impact in both physical functioning and fatigue/energy compared to male patients (Table 3).

Bettoni et al. [24] reported a positive relation of moderate magnitude between self-care and several QoL subscales (symptoms/problems, cognitive functioning, physical function, emotional well-being, energy/fatigue, depression, and anxiety). These results are similar to our findings, which reinforces the need for health professionals, including nurses, to provide interventions aimed at both the patient and the primary caregiver regarding their knowledge of HD, HD functioning, medication intake difficulties, allowed and prohibited foods, fluids intake, venous access care, prevention of HD symptoms, leisure, interpersonal relationships, HD complications, anti-coagulation treatments, the practice of emotional activities, and association with social groups.

We observed that the disease load, emotional state, and social support were the main factors related to self-care. Among many factors, these correlations could be explained not only by KF itself and HD but also by patients’ perception of the arterio–venous fistula. According to Silva et al. [25], venous access through a fistula leaves marks that alter the body’s aesthetics, making the body imperfect. These changes cause low self-esteem and attract the look of others. This gives discomfort to HD patients, who try to hide their fistula and at the same time recognize the fistula as essential for their own life. From this perception arises fear in the patient, as a catalyst for self-care. We found not gender differences in the vascular access type in the sample of the present study. However, previous studies report that gender female patients starting chronic HD are less likely to have access to an arteriovenous fistula than men, and females with an arteriovenous fistula have a higher rate of 1-year survival than those who do not have this vascular access [26]. Therefore, understanding the potential effect of this gender disparity on self-care and quality of life warrants further studies.

Previous works have documented the influence of variables that promote self-care behavior during HD, including knowledge [27], attitudes, ability, previous self-efficacy, motivation, higher scholarly, having a stable job, and a smaller number of HD sessions [28]. The nursing staff must consider these factors to develop nursing consultation so to improve therapeutic adherence and self-care, mainly focused on diet and medication and linked to a long involvement in the treatment and a more frequent contact with the primary caregiver [29].

In our study, the patients with higher self-care perception were treated with HD or hemodiafiltration and required a longer commute from home to the HD unit. Probably, the perceived self-care is linked to their ability to organize and perform the actions needed to achieve a certain degree of performance (e.g., to be able to arrive on time despite a long commute), and therefore these beliefs influence their goal-oriented behavior, such as the choice of a task, the persistence, the effort, and the acquisition of abilities [30].

Previous work has reported that women treated with HD had higher self-care than men [18], while others have found similar self-care between men and women but a lower self-efficacy in female patients [17]. In most cases, the onset of a terminal disease such as KF requires prolonged treatment, and the main caregiver is a member of the family [31]. Lee et al. [32] showed that HD-treated patients with family caregivers reported higher self-care scores than those without a family caregiver and found a significant correlation between self-care and social support. When we evaluated such correlation separately by gender, the correlation was significant only for female patients (Table 4). Further, it is known that if the patient is a man, the caregiver is often a woman (his wife, daughter, or mother), but if the patient is a woman, the caregiver usually is her daughter or her mother [31]. Moreover, in Latin American countries such as Mexico, where culture influences the housekeeping process, most women who become ill continue performing their roles as mother, daughter, or wife, prioritizing the care of others before their own care [33]. Therefore, it is necessary that health professionals, especially the nursing staff, recognize their educational role and develop strategies to teach renal patients about self-care, converting them into protagonists of the therapeutic process, including their family. This will contribute to the implementation of safe practices regarding the compliance to food intake and fluid intake restrictions, the balance between rest and physical activity, and the self-care of the vascular access and the fistula (e.g., during blood pressure measurements, drug administration, and when carrying weight with the arm).

One study limitation is the relatively small sample of participants, who were consecutively enrolled in two HD centers. Further studies with larger randomly selected samples from multiple HD centers are needed to increase the external validity of our findings and to confirm the absence of significance in several results of the present study. Future studies could use our preliminary findings to estimate the sample size needed to warrant a given statistical power.

## 5. Conclusions

Women with KF treated with HD perceived a higher impact of HD and reported a lower QoL than men. Despite having a similar self-care agency, the self-care correlations with QoL and HD symptoms resulted to be different between men and women treated with chronic HD. Such differences may be important in future nursing interventions to improve self-care and QoL among KF patients.

## Figures and Tables

**Table 1:** Anthropometric characteristics, comorbidities, and laboratory results for 102 kidney failure (KF) patients treated with hemodialysis (HD) or hemodiafiltration. Data are shown as mean ± standard deviation, median (percentile 25–percentile 75), or absolute frequency (percentage).

Variables	Women (*N* = 47)	Men (*N* = 55)	*p*-Value
Age (years)	36.6 ± 10.7	39.3 ± 14.1	0.287
Body mass index (Kg/m^2^)	23.2 ± 6.5	23.9 ± 6.6	0.909
Diagnosis time (months)	5 (3–12)	6 (3–12)	0.886
HD vintage (months)	3 (2–5)	3 (1–6)	0.964
Glucose (mg/dL)	89 (76–110)	90 (74–106)	0.965
Hemoglobin (mg/dL)	8.75 ± 1.67	10.33 ± 2.10	<0.001
Creatinine (mg/dL)	8.71 ± 1.87	11.87 ± 3.42	<0.001
Blood urea nitrogen (mg/dL)	51.06 ± 19.08	61.83 ± 21.6	0.012
Uric acid (mg/dL)	5.94 ± 1.14	6.59 ± 1.51	0.023
Albumin (g/dL)	4.02 ± 0.25	4.12 ± 0.28	0.056
Potassium (mEq/L)	4.82 ± 0.62	4.70 ± 0.72	0.420
Sodium (mmol/L)	136.6 ± 3.2	137.8 ± 2.7	0.046
Calcium (mg/dL)	8.7 ± 0.8	8.7 ± 1.0	0.978
Phosphorus (mg/dL)	4.27 ± 1.69	5.02 ± 2.09	0.059
KF etiology			0.015
Diabetes mellitus	4 (8.5%)	5 (9.1%)	
Essential hypertension	2 (4.3%)	13 (23.6%)	
Autoimmune disease	7 (14.9%)	2 (3.6%)	
Other	34 (72.3%)	35 (63.6%)	
HD mode			0.418
Hemodiafiltration	21 (44.7%)	29 (52.7%)	
Hemodialysis	26 (55.3%)	26 (47.3%)	
Vascular access			0.437
Arterio–venous fistula	21 (46.7%)	31 (58.5%)	
Tunneled catheter	21 (46.7%)	18 (34.0%)	
Non-tunneled catheter	3 (6.7%)	4 (7.5%)	
Expecting renal transplant	21 (45.7%)	23 (41.8%)	0.699
Arterial hypertension	31 (70.5%)	35 (63.6%)	0.475
Diabetes mellitus	8 (17.0%)	9 (16.4%)	0.929
Cardiac disease	7(14.9%)	9 (16.4%)	0.839
Autoimmune disease	6 (13.0%)	1 (1.8%)	0.027
Previous surgery	1 (2.2%)	1 (1.9%)	0.909
Food of drug allergies	12 (25.5%)	6 (10.9%)	0.009
Hepatitis B vaccine	10 (21.3%)	11 (20.0%)	0.874
Full vaccination scheme	8 (17.0%)	12 (21.8%)	0.543
Previous hospitalization	13 (27.7%)	9 (16.4%)	0.167
Blood transfusion	17 (36.2%)	16 (29.1%)	0.446
Smoking	1 (2.1%)	6 (10.9%)	0.080
Alcohol use	2 (4.3%)	7 (12.7%)	0.133

**Table 2:** Demographic characteristics of the study participants. Data are shown as mean ± standard deviation, median (percentile 25–percentile 75), or absolute frequency (percentage).

Variables	Women	Men	*p*-Value
	(*N* = 47)	(*N* = 55)	
Monthly income (pesos)	4000 (2000–5000)	3200 (2000–5000)	0.878
Commute time to clinic (hours)	1.00 (0.75–0.62)	1.00 (0.66–0.74)	0.742
Commute expenses (pesos)	100 (53–238)	100 (58–150)	0.222
Education			0.986
Illiterate	1 (2.1%)	1 (1.8%)	
Primary/secondary school	25 (53.2%)	30 (54.5%)	
High school/College	21 (44.7%)	24 (43.6%)	
Marital status			0.399
Married or common-law	27 (57.4%)	27 (49.1%)	
Single, widow(er), divorced	20 (42.6%)	28 (50.9%)	
Work status			0.341
Unemployed	18 (40.0%)	14 (26.4%)	
Remunerated work	22 (48.9%)	33 (62.3%)	
Non-remunerated work	5 (11.1%)	6 (11.3%)	
Family structure			0.888
Alone	1 (2.1%)	2 (3.6%)	
Nuclear	38 (80.9%)	43 (78.2%)	
Extensive	8 (17.0%)	10 (18.2%)	
Role in the family			0.193
Father/mother	22 (47.8%)	18 (33.3%)	
Son/daughter	14 (30.4%)	23 (42.6%)	
Husband/wife	9 (19.6%)	6 (11.1%)	
Brother/sister	1 (2.2%)	4 (7.4%)	
Other	0 (0.0%)	2 (3.7%)	
House ownership			0.281
Own	23 (48.9%)	35 (63.6%)	
Borrowed	10 (21.3%)	10 (18.2%)	
Other	14 (29.8%)	10 (18.2%)	
Health insurance system			0.660
IMSS	32 (69.6%)	37 (67.3%)	
ISSSTE	1 (2.2%)	2 (3.6%)	
Seguro popular	5 (10.9%)	3 (5.5%)	
None/other	8 (17.4%)	13 (23.6%)	
Private health insurance	12 (25.5%)	14 (25.5%)	0.993

IMSS: Instituto Mexicano del Seguro Social, ISSSTE: Instituto de Seguridad y Servicios Sociales de los Trabajadores del Estado.

**Table 3:** Self-care, hemodialysis (HD) symptoms, and quality of life (QoL) of the study participants. Data are shown as mean ± standard deviation or median (percentile 25–percentile 75).

Variables	Women (*N* = 47)	Men (*N* = 55)	*p*-Value
Self-care	77.1 ± 10.8	77.5 ± 9.9	0.871
HD symptoms			
Intensity (points)	35.2 ± 21.8	22.2 ± 16.5	0.001
Prevalence (%)	12.7 ± 6.5	8.9 ± 5.1	0.001
QoL			
Total score	68.8 ± 13.6	73.4 ± 11.1	0.064
Symptoms	83.3 (75.0–89.5)	89.5 (79.1–93.7)	0.002
Disease effect	68.7 (62.5–81.2)	78.1 (68.7–90.6)	0.074
Renal disease load	50 (25–69)	50 (31–75)	0.688
Work status	50 (50–100)	50 (0–100)	0.721
Cognitive function	86.6 (66.6–100.0)	86.6 (73.3–100.0)	0.499
Social interaction quality	86.6 (66.6–100.0)	86.6 (66.6–93.3)	0.560
Sexual function	93.7 (56.2–100.0)	100.0 (65.6–100.0)	0.640
Sleep	56.6 (50–63.3)	60.0 (53.3–63.3)	0.114
Social support	83.3 (66.6–100.0)	83.3 (66.6–100.0)	0.543
Positive interaction with health staff	75.0 (62.5–100.0)	87.5 (75.0–100.0)	0.105
Patient’s satisfaction	83.3 (50.0–100.0)	83.3 (66.6–100.0)	0.608
Physical functioning	70 (40–80)	75 (60–90)	0.033
Physical role	50 (25–100)	75 (25–100)	0.446
Pain	68 (55–90)	88 (68–100)	0.039
Overall health	50 (45–65)	65 (50–75)	0.041
Emotional status	80 (64–92)	84 (76–96)	0.063
Emotional role	100 (67–100)	100 (100–100)	0.307
Social function	75 (63–100)	88 (63–100)	0.497
Fatigue/energy	60 (40–80)	70 (55–80)	0.044

**Table 4:** Spearman correlation coefficients for quality of life (QoL), self-care, and HD symptoms in 102 KF patients. Significant correlations (*p* < 0.05) are indicated with an asterisk (*).

	Self-Care		HD Symptoms Prevalence		HD Symptoms Intensity	
Variables	Women (*N* = 47)	Men (*N* = 55)	Women (*N* = 47)	Men (*N* = 55)	Women (*N* = 47)	Men (*N* = 55)
Total score	0.415 *	0.293 *	−0.539 *	−0.512 *	−0.580 *	−0.513 *
Symptoms	0.167	0.040	−0.660 *	−0.631 *	−0.757 *	−0.610 *
Disease effect	0.380 *	0.137	−0.391 *	−0.403 *	−0.350 *	−0.409 *
Renal disease load	0.463 *	0.228	−0.320 *	−0.294 *	−0.317 *	−0.331 *
Work status	0.030	0.037	−0.175	−0.171	−0.261	−0.120
Cognitive function	0.328 *	0.002	−0.396 *	−0.573 *	−0.485 *	−0.556 *
Social interaction quality	0.151	0.216	−0.416 *	−0.455 *	−0.497 *	−0.453 *
Sexual function	0.297	0.091	−0.112	−0.165	−0.240	−0.237
Sleep	0.155	0.375 *	−0.334 *	−0.363 *	−0.446 *	−0.390 *
Social support	0.295 *	0.158	−0.261	−0.173	−0.246	−0.114
Positive interaction with health staff	0.284	0.132	0.133	−0.082	0.159	−0.011
Patient’s satisfaction	0.340 *	0.021	0.062	0.003	0.113	−0.001
Physical functioning	0.297 *	0.270 *	−0.249	−0.233	−0.196	−0.204
Physical role	0.163	0.158	−0.349 *	−0.104	−0.347 *	−0.119
Pain	0.081	0.095	−0.546 *	−0.479 *	−0.564 *	−0.485 *
Overall health	0.273	0.069	−0.295 *	−0.132	−0.333 *	−0.157
Emotional status	0.297 *	0.189	−0.603 *	−0.468 *	−0.669 *	−0.469 *
Emotional role	0.048	0.158	−0.351 *	−0.268 *	−0.438 *	−0.298 *
Social function	0.210	0.181	−0.493 *	−0.377 *	−0.488 *	−0.392 *
Fatigue/energy	0.300 *	0.272 *	−0.552 *	−0.450 *	−0.575 *	−0.419 *

QoL: quality of life.

**Table 5:** Spearman correlation coefficients between self-care and ordinal variables. Significant correlation (*p* < 0.05) is indicated with an asterisk (*).

Variables	All Patients (*N* = 102)	Women (*N* = 47)	Men (*N* = 55)
Age (years)	0.058	0.009	0.093
Body mass index (Kg/m^2^)	−0.190	−0.113	−0.259
Diagnosis time (months)	0.051	0.104	−0.023
HD vintage (months)	−0.078	−0.086	−0.105
Hemoglobin (mg/dL)	−0.049	0.014	−0.061
Glucose (mg/dL)	−0.185	−0.257	−0.136
Creatinine (mg/dL)	−0.056	−0.227	0.032
Blood urea nitrogen (mg/dL)	−0.001	−0.115	0.123
Uric acid (mg/dL)	−0.121	−0.179	−0.062
Albumin (g/dL)	0.170	0.146	0.194
Potassium (mEq/L)	0.028	−0.139	0.163
Sodium (mmol/L)	0.043	0.119	0.024
Calcium (mg/dL)	−0.093	−0.072	−0.122
Phosphorous (mg/dL)	−0.081	−0.070	−0.108
Monthly income	−0.009	0.017	−0.044
Commute time to clinic (hours)	0.213 *	0.206	0.220
Commute expenses (pesos)	0.094	0.167	0.027
Prevalence HD symptoms	−0.050	−0.175	0.019
Intensity HD symptoms	−0.018	−0.081	0.003

**Table 6:** Self-care score compared by gender for the studied nominal variables.

Variable	Women	Men
	(*N* = 47)	(*N* = 55)
Diabetes Mellitus		
Yes	69.9 ± 17.5	77.7 ± 8.1
No	78.7 ± 8.6 ^a^	77.5 ± 10.4
Arterial hypertension		
Yes	75.4 ± 11.8	77.4 ± 10.2
No	80.6 ± 7.9	77.6 ± 9.7
Cardiac disease		
Yes	72 ± 11.7	75.9 ± 10.4
No	78 ± 10.62	77.8 ± 9.9
Allergy		
Yes	78.1 ± 13.8	74.3 ± 3.5
No	76.8 ± 9.8	77.8 ± 10.4
Previous hospitalization		
Yes	76.1 ± 12.0	80.7 ± 9.6
No	77.5 ± 10.5	76.8 ± 10.0
Previous transfusion		
Yes	76.4 ± 11.8	80.3 ± 7.8
No	77.5 ± 10.4	76.3 ± 10.5
Alcohol drinking		
Yes	75 ± 8.2	80 ± 7.6
No	77.2 ± 11	77.1 ± 10.2
Education		
Illiterate, primary or secondary	75.2 ± 13	77.1 ± 9.7
High school or college	79.5 ± 6.9	77.9 ± 10.4
Marital status		
Married/common-law partner	77 ± 10.4	78 ± 10.6
Single/widow(er)/divorced	77.3 ± 11.6	77 ± 9.4
Work status		
Remunerated work	73.1 ± 10.5	78.4 ± 10.1
Unemployed/Non-remunerated	75.5 ± 11.0	76 ± 10.1
House ownership		
Own	76.8 ± 14.1	76.1 ± 11.1
Borrowed	79.4 ± 6.8	79.4 ± 81.6
Other	76.1 ± 6.5	76.1 ± 8
Health insurance system		
Government agency	77.4 ± 10.06	76.7 ± 10.1
None/other	74.1 ± 14.3	79.8 ± 9.2
Private health insurance		
Yes	77.2 ± 9.8	79.1 ± 10.1
No	77.1 ± 11.3	76.93 ± 9.9
Hemodialysis mode		
Hemodiafiltration	80.6 ± 6.9	78.5 ± 7.8
Hemodialysis	74.3 ± 12.6 ^b^	76.3 ± 11.9
Vascular access		
Arterio-venous fistula	79.4 ± 9.6	78.1 ± 7.5
Tunneled catheter	74.7 ± 12.1	73.6 ± 12.4
Non-tunneled catheter	81.1 ± 13.0	86 ± 10.5

^a^ *p* < 0.05 vs. women with diabetes mellitus. ^b^ *p* < 0.05 vs. women treated with hemodiafiltration.

## Data Availability

The data presented in this study are available on request from the corresponding author.

## Supplementary Materials

The following are available online at https://www.mdpi.com/article/10.3390/ijerph182413022/s1, File S1: Completed STROBE checklist (S1); File S2: Results of the reliability analysis of the Spanish version of the Dialysis Symptoms Index (DSI).
