# Supplementary material for: Gender-Specific Differences in Self-Care, Treatment-Related Symptoms, and Quality of Life in Hemodialysis Patients
Source: Int J Environ Res Public Health. 2021 Dec 10;18(24):13022. doi: 10.3390/ijerph182413022 (PMC8701918; doi:10.3390/ijerph182413022)
Supplement: Supplementary file 1 [file ijerph-18-13022-s001.zip › SupplementaryMaterial_S2_ReliabilityAnalysis.pdf]

**Supplementary Material - S2**

# Results of the reliability analysis of the Dialysis Symptoms Index (DSI), Spanish language translation (N = 102 patients)

**Cronbach's Alpha Based on Standardized Items = 0.889**

TOTAL (30 items)

| Mean | Variance | Standard deviation | Number of items |
|---|---|---|---|
| 48.38 | 225.96 | 15.03 | 30 |

**Hotelling's T-Squared Test**

| Hotelling's T-Squared value | F | df1 | df2 | p-value |
|---|---|---|---|---|
| 219.70 | 5.48 | 29 | 73 | <0.001 |

F: F-test statistic; df: degrees of freedom

| Total intra-class correlation | | | | |
|---|---|---|---|---|
| Intra-class correlation | Lower Bound | Upper Bound | F Test with True Value = 0 | p-value |
| 0.896 | 0.865 | 0.923 | 9.65 | <0.001 |
